# Temporal discounting and smoking cessation: choice consistency predicts nicotine abstinence in treatment-seeking smokers

**DOI:** 10.1007/s00213-020-05688-5

**Published:** 2020-11-20

**Authors:** Charlotte M. Grosskopf, Nils B. Kroemer, Shakoor Pooseh, Franziska Böhme, Michael N. Smolka

**Affiliations:** 1 Department of Psychiatry and Neuroimaging Center, Technische Universität Dresden, 01187 Dresden, Germany; 2 Department of Psychiatry and Psychotherapy, University of Tübingen, 72076 Tübingen, Germany; 3 Freiburg Center for Data Analysis and Modeling (FDM), University of Freiburg, 79098 Freiburg, Germany

**Keywords:** Choice consistency, fMRI, Intertemporal choice, Relapse, Smoking, Tobacco

## Abstract

**Introduction:**

Smokers discount delayed rewards steeper than non-smokers or ex-smokers, possibly due to neuropharmacological effects of tobacco on brain circuitry, or lower abstinence rates in smokers with steep discounting. To delineate both theories from each other, we tested if temporal discounting, choice inconsistency, and related brain activity in treatment-seeking smokers (1) are higher compared to non-smokers, (2) decrease after smoking cessation, and (3) predict relapse.

**Methods:**

At T1, 44 dependent smokers, 29 non-smokers, and 30 occasional smokers underwent fMRI while performing an intertemporal choice task. Smokers were measured before and 21 days after cessation if abstinent from nicotine. In total, 27 smokers, 28 non-smokers, and 29 occasional smokers were scanned again at T2. Discounting rate *k* and inconsistency var(*k*) were estimated with Bayesian analysis.

**Results:**

First, *k* and var(*k*) in smokers in treatment were not higher than in non-smokers or occasional smokers. Second, neither *k* nor var(*k*) changed after smoking cessation. Third, *k* did not predict relapse, but high var(*k*) was associated with relapse during treatment and over 6 months. Brain activity in valuation and decision networks did not significantly differ between groups and conditions.

**Conclusion:**

Our data from treatment-seeking smokers do not support the pharmacological hypothesis of pronounced reversible changes in discounting behavior and brain activity, possibly due to limited power. Behavioral data rather suggest that differences between current and ex-smokers might be due to selection. The association of choice consistency and treatment outcome possibly links consistent intertemporal decisions to remaining abstinent.

**Supplementary Information:**

The online version contains supplementary material available at 10.1007/s00213-020-05688-5.

## Introduction

The concept that a reward’s value decreases when being delivered with delay is described as temporal or delay discounting (Mischel, 1966; Stevens, 1975). Economic decision theory posits that the choice between two outcomes is based on the utility or value of each offer (Neumann and Morgenstern 1944). For instance, who would not choose €10 immediately over €10 in 1 year? But what if the later amount was €11 or even €100 in 1 year? For each offer, one needs to trade off the delay’s and the reward’s magnitude in order to estimate its subjective value (Kable and Glimcher, 2007). Moreover, across a set of choices, differences in how consistently future rewards are evaluated become apparent (Ripke et al., 2015, 2012), but the psychological relevance of choice consistency is currently not fully understood.

A preference towards immediate rewards has been reported in a number of pathological conditions. Especially substance use disorders (SUDs) are associated with elevated temporal discounting. Smoking (Bickel, Odum, and Madden, 1999; MacKillop et al., 2012; Reynolds, 2004), alcoholism (Mitchell, 2011; Odum and Rainaud, 2003), and illicit drug addiction (Kirby, Petry, and Bickel, 1999; Madden, Petry, Badger, and Bickel, 1997) are associated with a steeper discounting of future outcomes (cf. Amlung et al. 2017 for meta-analysis). This is commonly interpreted as an incapability to consider future effects on health when offered immediate enjoyment instead (Story, Vlaev, Seymour, Darzi, and Dolan, 2014).

Cross-sectional studies demonstrated that current smokers show steeper discounting compared to non-smokers and ex-smokers (Baker, Johnson, and Bickel, 2003; Bickel et al., 1999; Odum, Madden, and Bickel, 2002). This has often been interpreted as a pharmacological effect of cigarette consumption, possibly via nicotine, which might increase delay discounting (Reynolds, 2004). Nevertheless, longitudinal and experimental studies so far did not provide strong support for this “pharmacological hypothesis”: Short-term tobacco abstinence was reported not to influence temporal discounting of monetary rewards in dependent smokers (Mitchell, 2004; Yoon, Higgins, Bradstreet, Badger, and Thomas, 2009). In a previous study from our group, a single dose of nicotine only affected brain signals, but not discounting, in non-smokers (Kobiella et al., 2014). As a meta-analysis did not render conclusive results regarding changes during abstinence (Hughes, Dash, and Callas, 2015), calls for more longitudinal studies arose (Barlow, McKee, Reeves, Galea, and Stuckler, 2016; Cosgrove, 2016; Farabee, Schulte, Gonzales, and Grella, 2016), so that the effect of smoking cessation on temporal discounting would become clear.

An alternative interpretation of higher discounting in current smokers compared to non-smokers and ex-smokers are a priori existing differences and self-selection. Thereby, adolescents and young adults with steeper discounting have a higher probability to start smoking, and smokers with steep discounting then have a lower chance to quit again. In line with this *selection hypothesis*, Reynolds et al. reported that discounting in teenagers corresponds to trying cigarette smoking and facilitates smoking acquisition (i.e., to start smoking regularly)(Reynolds, Karraker, Horn, and Richards, 2003). Ultimately, the pharmacological and the selection hypotheses reflect the fundamental issue of cause or consequence that is key to a better understanding of addiction.

The discounting rate *k* estimated by fitting numerous binary choices to a hyperbolic value function (Mazur, 1987) does not fully describe intertemporal decision-making behavior. Another important characteristic of intertemporal choices is their consistency, which characterizes how strongly choices of an individual are driven by the difference between the values of both options. Some subjects may change their preferences sharply based on small value differences while others show no clear preference for the more valuable option, i.e., show a more random or inconsistent choice behavior. Yet most traditional models do not include a second parameter for choice consistency. It has been shown that participants behave differently in terms of consistency as estimated using the softmax decision function (Hare, Hakimi, and Rangel, 2014; Yechiam, Busemeyer, Stout, and Bechara, 2005) or by constructing receiver operating characteristic curve analyses for the set of choices by individual subjects and using the area under the curve (AUC)(Ripke et al., 2015, 2012). Collectively, these studies highlight the importance of extending the focus beyond the discounting rate *k* to better understand temporal discounting and its potential role in mental disorders.

On the neuronal level, there is converging evidence about brain networks coding the value of options, as well as underlying processes of top-down control, decision-making, and prospection (Kable and Glimcher, 2007; McClure, Laibson, Loewenstein, and Cohen, 2004; Peters and Büchel, 2011; Ripke et al., 2012) integrating information about amount and delay of offers and finally resulting in a choice for either the immediate or delayed reward. Importantly, some regions such as the value-tracking ventral striatum and ventromedial prefrontal cortex, or control regions in the dorsolateral prefrontal cortex, are densely dependent on dopaminergic neuromodulation that is known to be altered in smokers (Dagher et al., 2001; Fehr et al., 2008). In line with this, we previously found decreased reactivity of the ventral striatum of smokers compared to non-smokers during intertemporal choice, and an effect of acute nicotine administration on processing of the reward magnitude (Kobiella et al., 2014). Our group also demonstrated that lower consistency during intertemporal choice is associated with less brain activity in a fronto-parietal network (Ripke et al., 2012) and that the BOLD response in this network mediates the association between intelligence and choice consistency (Ripke et al., 2015). Therefore, we hypothesized a lower reactivity of the ventral striatum in smokers compared to controls before treatment. Moreover, in line with the pharmacological hypothesis, we expected increased reactivity after smoking cessation.

The aim of this study was to test whether the difference between current smokers and non-/ex-smokers can be better explained by the pharmacological or the selection hypothesis. For this purpose, intertemporal choice behavior (discounting rate *k* and consistency) and the respective neural correlates were assessed twice during fMRI in a group of smokers enrolled in behavioral treatment and two control groups (occasional smokers and non-smokers). For smokers in treatment, the second assessment was scheduled 3 weeks after cessation. Differences in discounting behavior and its neural underpinnings between smokers before cessation and controls would be in line with predictions from both competing hypotheses. The pharmacological hypothesis would predict that these readouts changed after smoking cessation. We would expect to see changes in smoker’s behavior or activation patterns. Contrariwise, the selection hypothesis would predict that discounting behavio, and neural correlates before cessation were associated with increased risk for relapse but did not change after cessation.

## Methods

### Procedure

Initially, prospective participants were screened for mental disorders using the Structured Clinical Interview for the Diagnostic and Statistical Manual of Mental Disorders, 4th edition (DSM-IV) and had no history of schizophrenia, bipolar disorder, SUD except cigarette smoking (lifetime), or other neurological or mental disorders (e.g., no depression in the last 12 months). The assessment included the Fagerström Test for Nicotine Dependence (FTND) (Heatherton, Kozlowski, Frecker, and Fagerström, 1991), Cigarette Dependence Scale (Etter, 2005), and questions regarding their smoking acquisition and education (see Table 1). All subjects were scanned at the Neuroimaging Center of TU Dresden. Prior to the investigation, all participants were informed about the experimental procedures. They received a monetary compensation for their participation together with the effective payment of one randomly selected intertemporal choice from their decisions (e.g., €30 transferred to their bank account with 60 days of delay). The study was approved by the institutional Ethics Committee of the Technische Universität Dresden and written informed consent was obtained from each participant in accordance with the Declaration of Helsinki.

**Table 1:** Group characteristics

	Controls		Smokers		
	Nonsmokers *n*(T1) = 29; *n*(T2) = 28	Occasional smokers *n*(T1) = 30 *n*(T2) = 29	All *n*(T1) = 44; *n*(T2) = 27	Abstainers *n*(T1) = 25; *n*(T2) = 21	Relapsers *n*(T1) = 19; *n*(T2) = 6
Variable	Mean (SD)	Mean (SD)	Mean (SD)	Mean (SD)	Mean (SD)
Age	38.4 (9.1)	35.6 (8.3)	37.3 (9.5)	38.3 (9.3)	35.9 (9.4)
Female	15 [52%]	15 [50%]	21 [48%]	11 [44%]	10 [53%]
BMI	24.5 (3.0)	23.3 (3.2)	25.1 (3.7)	24.4 (3.7)	26.0 (3.3)
Smoking behavior and nicotine consumption					
FTND	–	0.1 (0.4)	5.0 (2.0)	4.6 (2.1)	5.5 (1.7)
CDS	–	19.7 (4.2)	46.0 (6.8)	44.6 (6.7)	47.0 (6.6)
Cigarettes per day	–	0.80 (1.0)		18.6 (6.4)	19.4 (5.4)
Daily nicotine consumption (onset age in years)	–	–	16.7 (4.4)	16.8 (3.3)	16.7 (5.4)
Nicotine addiction (DSM-4, onset age in years)	–	–	19.0 (6.9)	19.7 (7.2)	18.1 (6.1)
Education					
Secondary school leaving certificate	7 [24%]	5 [17%]	18 [41%]	13 [52%]	5 [26%]
Higher education entrance qualification	22 [76%]	25 [83%]	26 [59%]	12 [48%]	14 [74%]
Professional qualification					
None/professional education	10 [34%]	8 [27%]	20 [45%]	14 [56%]	6 [32%]
Polytechnic/university degree	19 [66%]	22 [73%]	24 [55%]	11 [44%]	13 [68%]
Delay discounting					
T1 *k*	− 4.38 (1.4)	− 4.06 (1.4)	− 3.98 (1.2)	− 3.88 (1.2)	− 4.13 (1.3)
T1 *β*	− 1.28 (1.4)	− 0.65 (1.6)	− 1.01 (1.5)	− 0.80 (1.0)	− 1.28 (1.8)
T1 var(*k*)	0.26 (0.3)	0.22 (0.3)	0.32 (0.6)	0.14 (0.2)	0.56 (0.8)
T2 *k*	− 4.47 (1.7)	− 4.22 (1.7)	− 4.18 (1.3)	− 3.97 (1.2)	− 4.83 (1.7)
T2 *β*	− 0.03 (1.9)	− 0.22 (1.4)	− 0.34 (1.5)	− 0.45 (1.1)	− 0.02 (2.3)
T2 *var*(*k*)	0.20 (0.3)	0.13 (0.2)	0.11 (0.1)	0.11 (0.1)	0.11 (0.1)
Days between T1 and T2	25.6 (6.7)	31.6 (20.5)	27.6 (5.9)	27.7 (5.7)	27.5 (7.1)

In this table, *k* and *β* followed natural log-transformation

### Participants

**Fig. 1:**
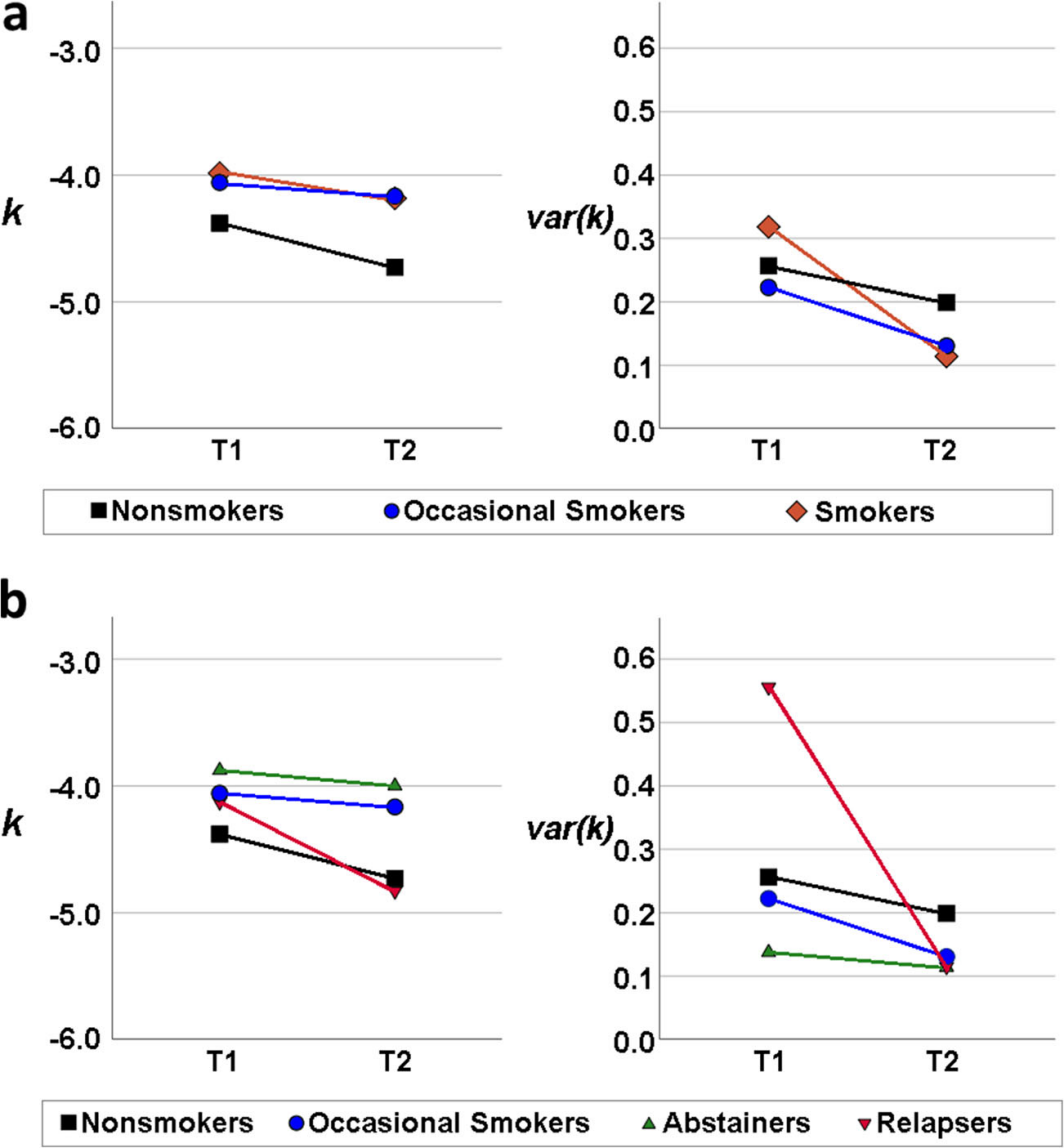
Mean *k* and mean var(*k*) of all groups at T1 and T2. All values following natural log-transformation. **a** No significant group differences between treatment-seeking smokers (brown) and controls (black: non-smokers, blue: occasional smokers) of delay discounting rate *k* were found. Smoking cessation (mean_abst_ = 21 days) did not significantly influence discounting at T2 despite an overall tendency for lower *k* at T2. Yet, one limitation is that smokers who relapsed before their second appointment were not invited and therefore could not be included into T2 group analysis. There were no differences in inconsistency var(*k*) between treatment-seeking smokers and controls. **b** Baseline discounting rate *k* of smokers relapsing within 30 days (red: relapsers) was not higher compared to those who abstained (green: abstainers). There were no differences in var(*k*) between treatment-seeking smokers and controls at T1. Yet var(*k*) at T1 was higher in relapsers (red) compared to abstainers (green)

We invited 44 smokers, 29 non-smokers (lifetime cigarette consumption < 20), and 30 occasional smokers (cigarette consumption < 5/week, and no nicotine dependence lifetime) to a baseline assessment (T1). The latter two groups served as control groups (Fig. 1, Table 1). All participants were right-handed. Dependent smokers took part in a widely offered manual-based cognitive behavioral group therapy program in Germany (“Rauchfrei Programm,” Wenig et al. 2013) and were scanned in a “smoking as usual” state before quitting and 2 to 5 weeks (21.0 ± 4.9 days) after quitting smoking for follow-up (T2). Breath carbon monoxide tests were used to verify abstinence of smokers at follow-up. Control groups were invited for a follow-up scan as well. At T2 we collected complete behavioral and fMRI data sets of 28 non-smokers, 29 occasional smokers, and 27 smokers in treatment. We excluded 13 smokers, who relapsed before their T2 appointment, as drop-outs, as well as 4 smokers who remained abstinent until T2 but did not want to participate in another fMRI scan. In our control group, one non-smoker and one occasional smoker dropped out as they declined a T2 fMRI scan.

### Behavioral data

#### Treatment outcome

For further analysis, dependent smokers were classified as *relapsers* if they relapsed early during the active treatment which ended approximately 30 days after the attempt to quit smoking, and as *abstainers* if they did not relapse within the first month. We chose this mid-term abstinence as one outcome to obtain comparable subgroup sizes and thereby maximize power for statistical analyses of behavioral and imaging data. Additionally, time to relapse beyond the first 30 days was further assessed via personal follow-up interviews after 3 and 6 months and inquiries via phone. Relapse was generally defined as smoking on more than 1 day. Therefore, smoking on 1 day, followed by abstinence for all subsequent days, would be considered a “lapse.” The same definition was communicated in the cognitive behavioral therapy program (Wenig et al., 2013).

#### Intertemporal choice task

All participants underwent two fMRI sessions (T1 and T2) performing an intertemporal choice task which we optimized for fMRI application (Kable and Glimcher, 2007) and described in earlier publications (Ripke et al., 2015, 2012). Briefly, prior to each scan, participants performed a calibration session. During this pre-scan training, the discounting parameter *k* of each participant was individually assessed. In each trial, participants had to choose between the immediate amount 20 € and a larger but later amount. The first three trials were not included in the adaptation so that subjects could practice the task. Then, 50 intertemporal decision trials were presented with 5 different delay levels (10, 30, 60, 120, or 180 days). The delay level changed after 10 decisions. At all five delay levels, the size of the first amount presented was 40 €. The pre-scan test included an adaptive staircase algorithm: offered amounts of money were raised or reduced depending on the previously made decision. If the delayed amount was chosen, the next trial included a delayed amount, which was reduced by half the difference between immediate and delayed reward, and vice versa. The indifference points were estimated as the mean of the maximum delayed amount rejected and the minimum delayed amount chosen. After finding the indifference amount for each delay, these points were fit to a hyperbolic function using ordinary least squares. Thereby, we characterized the subjective value *V* of a reward across the range of delays.

1 $$ V\left(A,d\right)=\frac{A}{\left(1+k\times D\right)} $$

Here, *k* represents the hyperbolic discounting rate, *A* represents the amount of money, and *D* the delay in days. We used Presentation® software (Version 14.0, Neurobehavioral Systems Inc., Berkeley, CA, USA) and MATLAB R2015a (The MathWorks Inc., Natick, MA, USA) to perform the paradigm.

The fMRI task consisted of 90 trials with the same delays as in the pre-scan session (10, 30, 60, 120, 180 days). The offers were adapted to the previously assessed *k* value in a way that (1) subjects were expected to choose the immediate reward in half of the trials, (2) the mean value of all delayed rewards would be the same (30 €) for each subject, and (3) the maximal value of all rewards was twentyfold the minimum value (Ripke et al., 2012). For each of the 5 delays, we computed 18 values (9 higher than the individual immediate amount and 9 lower than the individual immediate one) using the following formula:

2 $$ {V}_d={V}_0\times \frac{1+\left(k\times D\times c\right)}{1+\left(k\times D\right)} $$

To ensure that for all values (*V*_*d*_) lower than the immediate reward the respective delayed amounts were higher than the immediate ones, we included the parameter *c* and set it to 0.1, 0.15, 0.2, 0.25, 0.3, 0.35, 0.4, 0.45 and 0.5 respectively. The maximum value (*V*) for each delay was exactly twentyfold the lowest value for the minimum value for each subject, i.e., the 180 days (*D*) condition. The difference between immediate reward and maximum reward value was divided into 9 equidistant value categories above the immediate reward. The mean of all computed values (*V*) was standardized to 30 € and the immediate amount was adapted for each participant so that rewards were presented with the same mean over all trials for each subject. Finally, all respective amounts (*A*) were computed using the formula:

3 $$ A=V+\left(V\times k\times D\right) $$

Prior to the fMRI scan, all 90 offer pairs were calculated as described above and randomly mixed. The immediate amounts were not presented during the task but if the immediate reward was chosen, the immediate amount was shown in the feedback. At the beginning of each trial, participants were presented an offer incorporating an amount of money and its delay to receipt for 2 s. This presentation epoch was followed by a 4-s fixation cross. Next, participants had 2 s to select the preferred option. To choose the later reward, participants had to press the button on the side of the exclamation mark and vice versa for the immediate reward. The side of the screen where the exclamation mark appeared was randomized to avoid potential confounds of motor laterality. Feedback about the chosen amount was provided immediately after the button press. The whole task duration was 25 min.

#### Bayesian framework for individual trial-based analysis

To assess an individuals’ consistency in intertemporal choice tasks, approaches using parameters such as the “percentage of the same option choice for a given *k* value” (Cho et al., 2010), AUC curve analysis (Ripke et al., 2012), or the inverse temperature of the softmax likelihood function *β* (Peters, Miedl, and Büchel, 2012; Pooseh, Bernhardt, Guevara, Huys, and Smolka, 2018) have been reported before. For our analyses, apart from a hyperbolic value function, we used a softmax decision function (predicting decision probabilities depending on value differences), so that consistency and discounting rate could be estimated together.

Specifically, we used Bayesian analysis to characterize the performance of the participant. The prior belief about the value of *k* and the inverse temperature *β* (a measure of choice consistency) was adjusted for every trial. Thereby, we calculated the decision probabilities for each trial based on our informative model which included the hyperbolic discounting function (Eq. 1) and the softmax decision function (Eq. 4, see below), as described by Pooseh et al. (2018).

4 $$ P(A)=\frac{e^{\beta \times V(A)}}{e^{\beta \times V(A)}+{e}^{\beta \times V(B)}} $$

Pre-scan *k* entered this analysis as a prior for the trials in the fMRI scanner. The parameter *β* represents the slope of the softmax decision function that controls the degree to which decisions can be accurately predicted.

Using this model, we could predict choices for any given *k* and *β*. Hence, *P*(*A*_*t*_| *k*, *β*) is known at every trial. However, we were interested in estimating *k* and *β* given the observed choices *P*(*k*, *β*| *A*_*t*_). In order to connect the two probabilities, we used the Bayes’ rule (Birnbaum, 1962).

5 $$ P\left(k,\beta |{\mathrm{A}}_{\mathrm{t}}\right)=\frac{P\left({\mathrm{a}}_{\mathrm{t}}|k,\beta \right)\times P\left(k,\beta \right)}{P\left({A}_t\right)} $$

Thereby, the posterior distribution on parameter values is proportional to the product of the likelihood of the observation and the prior belief on the parameters. We discretize the parameter space *R* into an equally spaced 2D region with *k*_min_ ≤ *k* ≤ *k*_max_ and *β*_min_ ≤ *β* ≤ *β*_max_ . In this setting the Bayes’ rule simplifies to

6 $$ P\left(k,\beta |A\right)=\frac{1}{Z}P\left(A| k\beta \right)\times P\left(k,\beta \right) $$

where the factor 1*/Z* normalizes the product over the discrete domain *R*. We assume that the two parameters are independent and expect them to be skewed, i.e., approximately lognormal (Lovric, 2011). The independence assumption lets us build a joint likelihood distribution *P*(*k*, *β*) for the Bayesian framework.

Given our Bayesian framework, we are provided with the estimation of *k* and *β* together with the variance of *k* and *β*. The variance of *k* at the end of the task, var(*k*), can be understood as the width of the posterior likelihood distribution of the estimated *k*. During the task, the variability will increase after an unexpected choice, but it should decrease over the course of the experiment following consistent choices. Using the final estimate of this parameter, we can assess how consistently a participant acts across consecutive choices.

Within our behavioral model, two parameters are directly related to the consistency of choices, namely *β* and var(*k*)*.* However, the two parameters are complementary and based on different theoretical perspectives: *β* is an estimated parameter in the softmax function indicating how strongly a subject’s decision is determined by increasing or decreasing the offer’s subjective value. In contrast, var(*k*) is derived from the dynamic Bayesian estimation over the course of the task. It captures an incongruence of a decision with the current expected discount rate and increases whenever incongruent decisions are made and *k* has to be adjusted accordingly. In other words, when incongruent decisions are observed, it becomes more difficult from a computational perspective to capture all decisions with one discount rate, which is reflected in an increased uncertainty of the “true” value. Thus, var(*k*) represents the “fickleness” of intertemporal choice across trials. Intriguingly, low *β* values can both occur with relatively low and high var(*k*), but high *β* values preclude high var(*k*). To normalize the distributions, we refer to *k* and *β* following natural log-transformation.

### Imaging data

Image registration was performed using a 3-T whole-body MRI scanner (Magnetom TRIO, Siemens, Erlangen, Germany) with a standard 12-channel head coil. Head stabilization was attained using additional tomograph equipment pillows. For functional data registration, gradient echo-planar imaging (EPI) with 2.41 s of repetition time (TR), 25 ms of echo time (TE), and a flip angle of 80° was employed. A total of 642 whole brain scans were acquired from 42 transversal slices (2-mm thickness, 1-mm gap), aligned axially with 30° to the anterior commissure–posterior commissure line, with a 192-mm field of view (FOV), 64 × 64 pixels matrix size, and a 3 × 3 × 2 mm^3^ voxel size. For structural abnormality checking, a T1-weighted anatomical 3D magnetization–prepared rapid gradient echo (MPRAGE) dataset was acquired (TR = 1.90 s, TE = 2.26 ms, FOV = 256, 176 slices, 1 × 1 × 1 mm^3^ voxel size, flip angle = 9°).

SPM12 (Wellcome Department of Cognitive Neurology, London, UK) in MATLAB R2015a was used for preprocessing and statistical analyses. The acquired images followed slice-time correction, spatial realignment, and normalization to the standard MNI (Montreal Neurological Institute, Quebec, Canada) EPI template and resampled (voxel size: 3 × 3 × 3 mm^3^). Smoothing was performed using an isotropic Gaussian kernel (8 mm FWHM). We excluded data sets from 5 participants at T1 (and consequently at T2) due to excessive head movement (2-mm threshold) and one participant because normalization failed.

For the general linear model (GLM), we included five first-level regressors of the task: offer presentation onset, offered subjective value (as a parametric modulator), motor response left, motor response right, and missing responses. Therefore, we assessed both value-independent (first regressor) and value-dependent (second regressor) processing of offers. In line with previous studies, the subjective value of each offer was computed according to pre-scan *k* (Ripke et al., 2015). Further, we entered the six realignment parameters as regressors of no interest to account for linear movement effects. Data was high pass filtered with 128 s as cutoff.

At the group level, we then tested for value-independent (at offer presentation) and value-dependent main effects during intertemporal choice. To identify possible differences between our participant groups, we included first-level contrast maps of non-smokers, occasional smokers, abstinent smokers (if abstinence > 30 days), and early relapsers into two second-level factorial designs (factor group, 4 levels). To test whether smokers differ from controls, we computed contrasts between smokers, non-smokers, and occasional smokers (question 1). To test whether brain responses are associated with risk for relapse, we compared abstaining and relapsing smokers (question 3). To identify changes after cessation, we set up second-level contrasts with first-level images (follow-up—baseline) for both contrasts (value independent and dependent) and compared abstaining smokers with controls (question 2). Primarily, thresholds for all statistical analyses at the group level were set at *p* = 0.001 uncorrected.

### Data analysis

Our above-mentioned behavioral variables were tested as follows: The temporal discounting rate *k* and var(*k*) were tested for normality using Shapiro-Wilk test. Groups were compared using Student’s *t* tests (*k*) or Mann–Whitney *U* tests for var(*k*) which was not normally distributed. We used ANOVA to test for longitudinal changes of *k* and Cox regression to test if *k* predicted relapse. As var(*k*) was not normally distributed, we performed Wilcoxon signed-rank test to compare T1 and T2 values.

## Results

### Temporal discounting rate

First, we compared the temporal discounting rate *k* in treatment-seeking smokers and controls. Contrary to the pharmacological and the selection hypotheses, discounting was not significantly higher in smokers in treatment (*M* = − 3.98, SD = 1.21) compared to non-smokers (*M* = − 4.38, SD = 1.43); *t*(71) = 1.27, *p* = 0.209, and also occasional smokers (*M* = − 4.06, SD = 1.43); *t*(57) = − 0.86, p = 0.392 at T1 (Fig. 1a, cf. Table 1). Additionally, groups did not differ significantly when assessed 21 days later at T2. Non-smokers’ *k* (*M* = − 4.73, SD = 1.68) at T2 was not lower than ex-smokers’ *k* (M = − 4.18, SD = 1.33); *t*(53) = − 1.33, *p* = 0.189 or occasional smokers’ *k* (*M* = − 4.16, SD = 1.65); *t*(55) = − 1.27, *p* = 0.208.

Second, there was no main effect of time (*F*(1,81) = 2.651, *p* = 0.107) and no interaction of time and group on discounting (*F*(2,81) = 0.193, *p* = 0.825), i.e., smoking cessation did not lead to changes in discounting.

Third, discounting rate *k* did not predict the time to relapse (HR = 1.06, Wald = 0.14, *p* = 0.71, 95% CI = 0.79–1.41) Fig. 1

### Choice consistency

Next, we assessed choice consistency var(*k*) as estimated from Bayesian trial-by-trial analysis. First, the distributions differed from normal distribution (Shapiro-Wilk *p* < 0.05); therefore, nonparametric tests were applied. There were no differences between var(*k*) in non-smokers (*M* = 0.26, SD = 0.06) and treatment-seeking smokers (*M* = 0.32, SD = 0.60), *U* = 576.0, *Z* = − 0.699, *p* = 0.485) or in occasional smokers (*M* = 0.223, SD = 0.33); *U* = 335.0, *Z* = − 1.516, *p* = 0.129 at T1 (cf. Fig. 1a). Neither did *β* differ between non-smokers (*M* = − 1.28, SD = 1.46) and treatment-seeking smokers (*M* = − 1.01, SD = 1.47), *t*(71) = − 0.79, *p* = 0.432, or occasional smokers (*M* = − 0.65, SD = 1.61), *t*(57) = − 1.58, *p* = 0.120.

Second, var(*k*) generally was lower at T2 than at T1 (*Z* = − 2.14, *p* = 0.033), indicating less inconsistency, which matched with lower *β* values *t*(83) = − 3.68, *p* = 0.001, as var(*k*) and *β* are inversely correlated. However, at T2, there was no significant difference between the non-smokers’ var(*k*) (*M* = 0.20, SD = 0.26) compared with ex-smokers’ var(*k*) (*M* = 0.11, SD = 0.11) (*U* = 311.00, *Z* = − 1.13, *p* = 0.259).

**Fig. 2:**
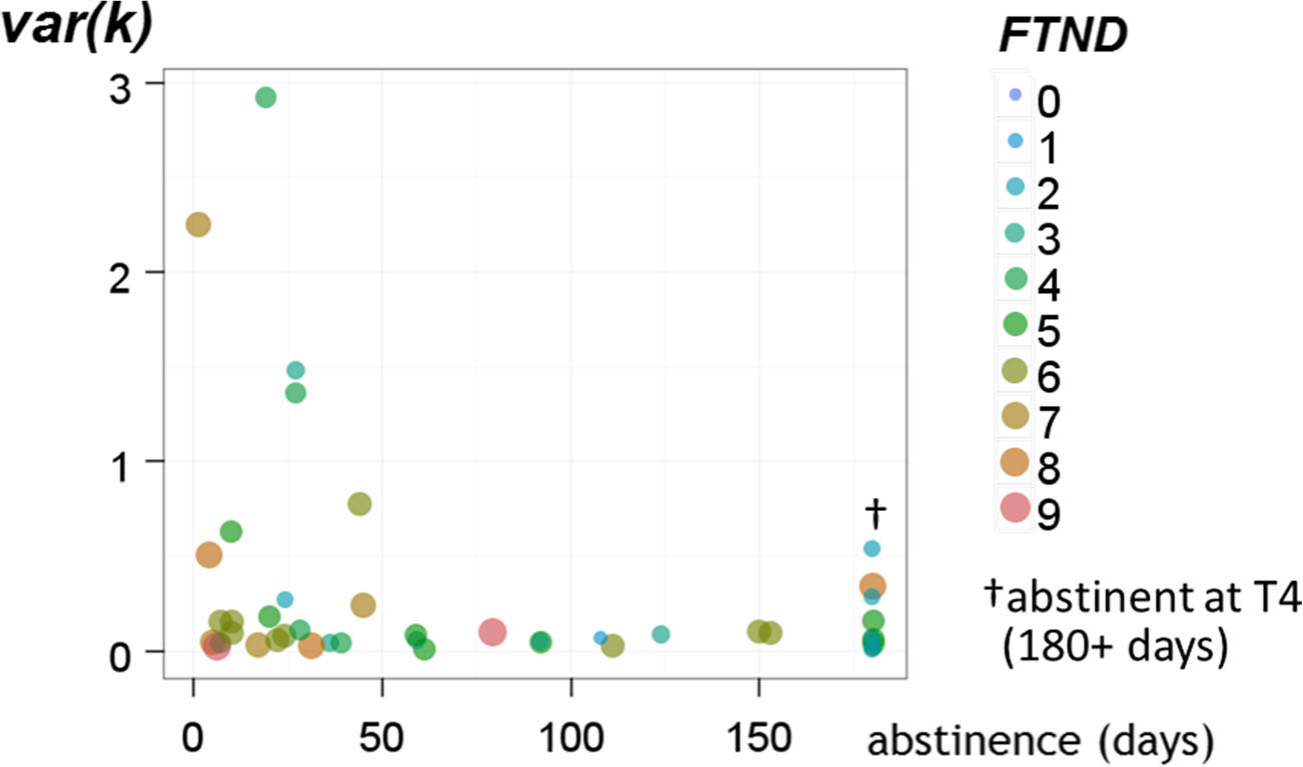
FTND (Fagerström Test for Nicotine Dependence), var(*k*) and period of abstinence. This plot displays the measured var(*k*) at T1 of all smokers against the duration of abstinence. Overall, it shows that subjects with higher var(*k*) tend to relapse earlier than subjects with lower var(*k*). FTND assessment of nicotine dependence predicted time to relapse. Especially low and very low nicotine dependence levels are associated with better treatment outcomes. Both factors contributed independently to Cox survival prediction. Smokers with high var(*k*) show earlier relapse regardless of their FTND scores. In this figure, var(*k*) followed natural log-transformation

Third, notably, var(*k*) at T1 in smokers relapsing within 30 days was higher compared to those smokers who abstained (*U* = 143.00, *Z* = 2.24, *p* = 0.025, Cohen’s effect size *r* = 0.33, Fig. 1b). In addition to the analysis of short-term outcome at the end of treatment (approximately 30 days after cessation), we used survival analyses to cross validate predictors for smoking relapse over a 6-month period. This included nicotine dependence as covariate as well as discounting behavior to predict relapse throughout the 180 days of follow-up. In accordance with the literature, nicotine dependence assessed by the FTND predicted time to relapse (HR = 1.217, CI = 1.03–1.42 *p* = 0.019). Yet as mentioned above, *k* did not predict time to relapse in our sample of smokers*.* Again, we found that var(*k*) predicted time to relapse, in addition to the FTND (Cox regression with 3 factors: *k* HR = 1.005, CI = 0.74–1.36, *p* = 0.976; FTND HR = 1.24, CI = 1.05–1.47, *p* = 0.011; var(*k*) HR = 2.18, CI = 1.23–3.82, *p* = 0.007), cf. Fig. 2. Taken together, these analyses indicate that var(*k*) is associated with both early and mid-term tobacco abstinence.

For further illustration of both *k* and var(*k*), boxplots of all groups at T1 and T2 are provided in the supporting information.

### Imaging data

#### Value-independent decision network

**Fig. 3:**
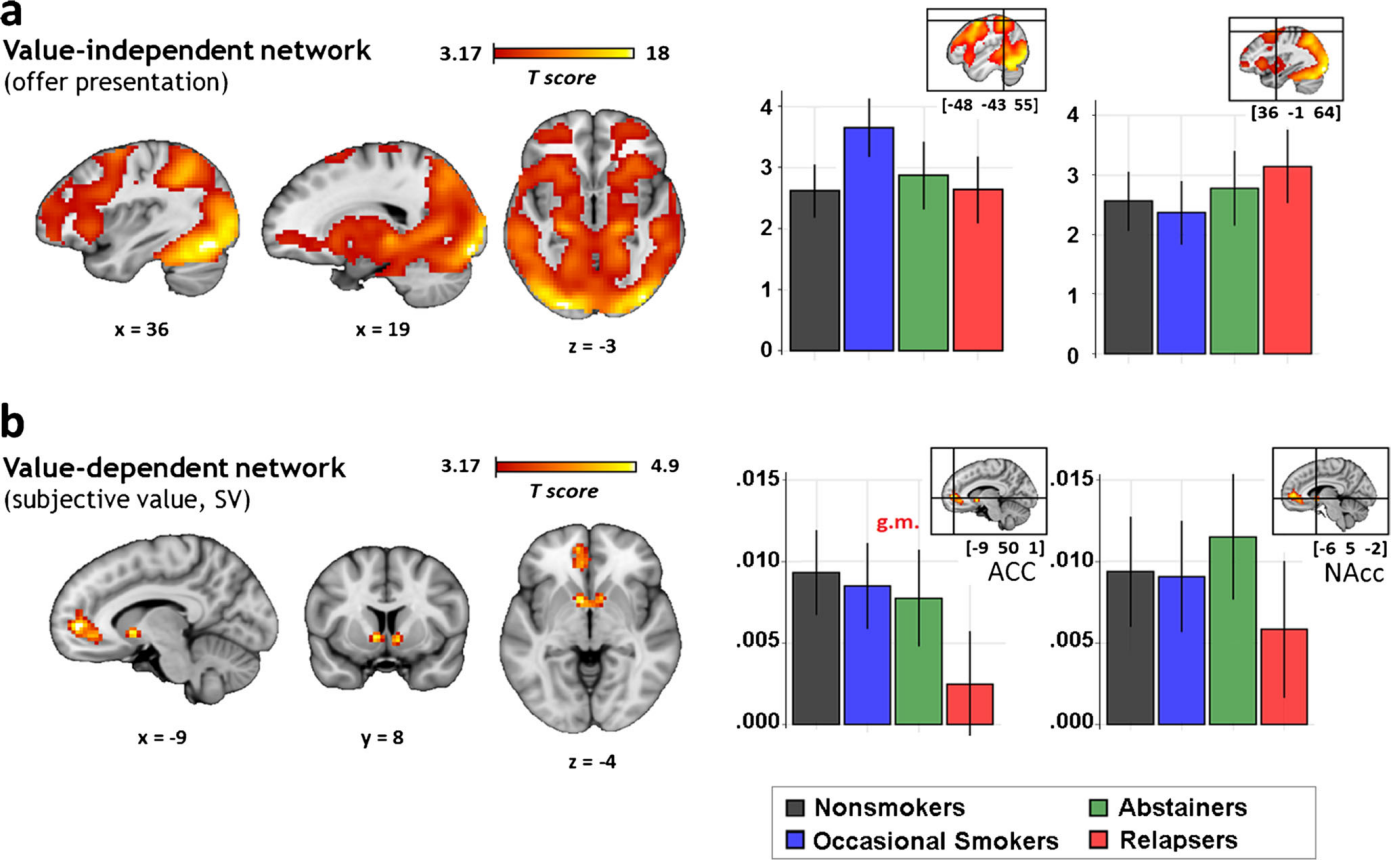
Main positive effects on BOLD response at T1. *p* < 0.001 uncorrected, *k* = 10, for illustration, clusters extracted with threshold *T* > 3.17, error bars indicate a 90% CI. **a** Value-independent network. During the offer presentation epoch, a broad increase in BOLD signal was observed in the hypothesized regions, including the dorsolateral prefronal cortex as well as parietal and occipital regions. Beta weight bar plots show activation in all groups. **b** Value-dependent network. High subjective value as a parametric modulator reflects in increased BOLD response in the striatal nucleus accumbens (NAcc) and anterior cingulate cortex (ACC). There are no significant differences in value tracking between groups and time (similar activation patterns at T2)

As reported in previous studies (Ripke et al., 2015, 2012), the offer presentation was reflected in a wide-ranging increase in BOLD response in the value-independent decision network with peak activation in occipital, parietal, and fronto-temporal areas. First, no differences between treatment-seeking smokers and non-smokers survived our whole brain threshold of *p* > 0. 001 at T1 (Fig. 3a). Second, smoking cessation did not significantly alter BOLD responses in this value-independent decision network, i.e., there was no interaction of group and time. Third, we did not find differences of the value-independent BOLD response between smokers relapsing during active treatment and abstainers (*p*_FWE-corr_ = 0. 962).

#### Valuation network

The subjective value of the delayed offers was tracked in the “valuation network”: high subjective value corresponded to an increased BOLD signal in the ventral striatum/nucleus accumbens, and perigenual anterior cingulate cortex (ACC, peak: − 9 mm × 50 mm × 1 mm, *T* = 4.97, *k* = 208, *p*_FWE-corr_ < . 001; NAcc peak: − 6 mm × 5 mm × − 2 mm, *T* = 4.86, *k* = 81, *p*_FWE-corr_ = 0.039; cf. Fig. 3b). First, there were no significant group differences between smokers and controls. Second, there were no interactions of time and group (smokers vs. controls) that survived correction for multiple comparisons at the whole brain or region-of-interest level of analysis, i.e., smokers did not show substantial differences in value tracking after smoking cessation. Third, there were no significant differences between relapsers and abstainers (*p*_FWE-corr_ > 0.81).

## Discussion

Temporal discounting is a well-established behavioral marker in addiction research (Bickel, Koffarnus, Moody, and Wilson, 2014). The aim of this study was to clarify whether differences reported between current smokers compared to non-smokers and ex-smokers are due to a priori existing differences that increase the propensity to develop and maintain tobacco dependence and “self-selection,” or whether, on the contrary, those differences are due to reversible neuropharmacological effects of ingredients of tobacco smoke.

We investigated whether temporal discounting, consistency of intertemporal choices, and corresponding brain signals (1) differ between smokers in treatment and controls, (2) change after smoking cessation, and (3) predict time to relapse. Contrary to previous studies, we did not find steeper discounting in our group of treatment-seeking smokers. Furthermore, the discounting rate *k* was neither affected by abstinence after smoking cessation nor was it associated with time to relapse. Nevertheless, lower consistency of intertemporal choices was a significant predictor of relapse. Therefore, fickleness in making intertemporal decisions emerged as an important complementary parameter.

In line with the behavioral results, the imaging results supported the absence of cross-sectional differences between treatment-seeking smokers and controls. We found similar activation patterns of value-independent and value-dependent networks during intertemporal choice as in earlier studies (Ripke et al., 2015, 2012) at T1, as well as at T2. Notably, we observed no significant changes after smoking cessation in our imaging data. However, when considering that our groups showed no behavioral differences such as steeper delay discounting to begin with, it appears coherent that the cerebral activation patterns are not significantly distinct either. Thus, our findings support the interpretation that the steepness and consistency of intertemporal choices are stable characteristics (Audrain-McGovern et al., 2009a), which are not strongly modulated by reversible pharmacological effects of nicotine (Kobiella et al., 2014) or tobacco.

Contrary to our prior expectation, we did not find steeper discounting in our group of treatment-seeking smokers compared to controls. At first glance, this appears to contradict a well-studied phenomenon which has been reported numerously (Bickel et al., 1999; MacKillop et al., 2011) but not exclusively (Clewett et al., 2014). We too found higher *k* values in non-treatment-seeking smokers compared to non-smokers in a prior study (Kobiella et al., 2014). However, in this study, we investigated only subjects who opted in for a cognitive behavioral treatment program which is offered every 4–6 weeks. When willing to participate, they were scheduled for our study’s assessments. Thereby, we possibly recruited a particular subgroup of smokers with good abilities in future-directed planning. In retrospect, we believe that this self-selected group of highly motivated smokers behaved more like non-smokers in terms of forward planning and might show lower temporal discounting than smokers in general. In line with this assumption, smokers with a higher intention to quit were reported to have lower discounting rates (Athamneh, Stein, and Bickel, 2017). Furthermore, a study by Audrain-McGovern and colleagues previously observed that non-treatment-seeking smokers show higher discounting than treatment-seeking smokers and ex-smokers (Audrain-McGovern et al. 2009b). It seems that propensity for tobacco dependence and a lower likelihood to seek treatment are associated with steep temporal discounting. Therefore, our finding is also in line with the selection hypothesis. Accordingly, all groups (smokers vs. controls, relapsers vs. abstainers) showed similar tracking of subjective value in the “valuation network” (Frost and McNaughton, 2017; Wesley and Bickel, 2014).

In addition to the general propensity to discount future rewards, consistency has received more widespread attention in the last decades regarding psychiatric and neurodegenerative disorders (Burton, Hultsch, Strauss, and Hunter, 2002; MacDonald, Li, and Bäckman, 2009; Troyer, Vandermorris, and Murphy, 2016), and specifically addiction (Liu et al., 2012). Measuring within-person variability in task performance therefore might bear meaningful information, in addition from the direct outcome measurements of the task. In this vein, we captured the consistency of intertemporal decisions by analyzing probabilities of value-based choices using Bayesian computation. Notably, variability in intertemporal choices was elevated in relapsers both in cross-sectional and survival analyses of relapse even after accounting for nicotine dependence. This aspect of consistency might be relevant in the light of smoking cessation because a lapse in future-directed choices may eventually lead to relapse. This, too, can be interpreted in favor of the selection hypothesis.

Given that not all relapsers displayed high var(*k*), there might be different factors which facilitate early relapse. In line with the current understanding of the development of compulsive behavior in addiction (Everitt and Robbins, 2016), inconsistency can be considered an endophenotype which might explain early relapse, independently from the well-described influence of nicotine dependence or the overall effect of heavy discounting in smokers provided by cross-sectional evidence.

Our study bears noteworthy limitations regarding the rejection of the pharmacological hypothesis. First, not having found significant differences does not prove their non-existence. The general problem of power and sample size are apparent in our study, which is, to our knowledge, the first longitudinal fMRI study with treatment-seeking smokers. Also, our sample of treatment-seeking smokers does not show higher delay discounting and therefore is probably not representative for smokers in general. We cannot exclude the possibility that smokers with higher temporal discounting might become more future directed after smoking cessation. Thus, we cannot generalize our finding of steady temporal discounting after smoking cessation. Second, we decided to investigate our participants only twice, at baseline and again after at least 14 days of smoking abstinence to minimize the effect of acute withdrawal symptoms (Hughes, 2007). Therefore, we cannot exclude the possibility of behavioral and neuronal changes after shorter or much longer times of abstinence. To facilitate multiple assessments after cessation, it might be insightful to use ecological momentary assessment (Waters and Li, 2008; Wilson, Smyth, and MacLean, 2014). Third, in terms of our fMRI data, we recognize that the power of our study is limited and therefore, we cannot rule out the possibility that there are small effects which we only failed to detect. Similarly, differences in brain activation might occur if groups showed substantial differences in delay discounting. Yet, as we published earlier (Fröhner, Teckentrup, Smolka, and Kroemer, 2019), value-dependent BOLD responses fail to report individual variance accurately due to low intra-individual reliability. Finally, in terms of inconsistency, another relevant limitation is poor effort. In almost every experimental task, the performance of participants is affected by their motivation when doing the task. Therefore, we cannot exclude the possibility that our measure of inconsistency is also confounded by poor effort. An alternative interpretation may be that subjects who are not strongly motivated or attentive to perform the task are less motivated to adhere to the treatment requirements and therefore more likely to relapse.

In summary, discounting in treatment-seeking smokers did not differ from non-smokers, was not associated with treatment outcome, and tobacco abstinence did not affect discounting behavior as well as related fMRI brain activity in smokers. Our data indicates that discounting behavior is not mainly due to nicotine. As a result, previously observed lower discounting rates in ex-smokers might mainly be due to self-selection or other effects but not a pharmacological effect. Interestingly, higher consistency of intertemporal choices predicted lower risk for relapse to smoking. As episodic future thinking was shown to have an effect on delay discounting (Peters and Büchel, 2010; Stein, Tegge, Turner, and Bickel, 2018), being consistent in one’s thoughts and decisions might be an important prerequisite for remaining abstinent and could also be of value for further treatment developments.

## Supplementary Information

ESM 1 (DOCX 53 kb)
