# Supplementary material for: Temporal discounting and smoking cessation: choice consistency predicts nicotine abstinence in treatment-seeking smokers
Source: Psychopharmacology (Berl). 2020 Nov 20;238(2):399–410. doi: 10.1007/s00213-020-05688-5 (PMC7826310; doi:10.1007/s00213-020-05688-5)
Supplement: Supplementary file 1 — (DOCX 53 kb) [file 213_2020_5688_MOESM1_ESM.docx]

**Supporting information**


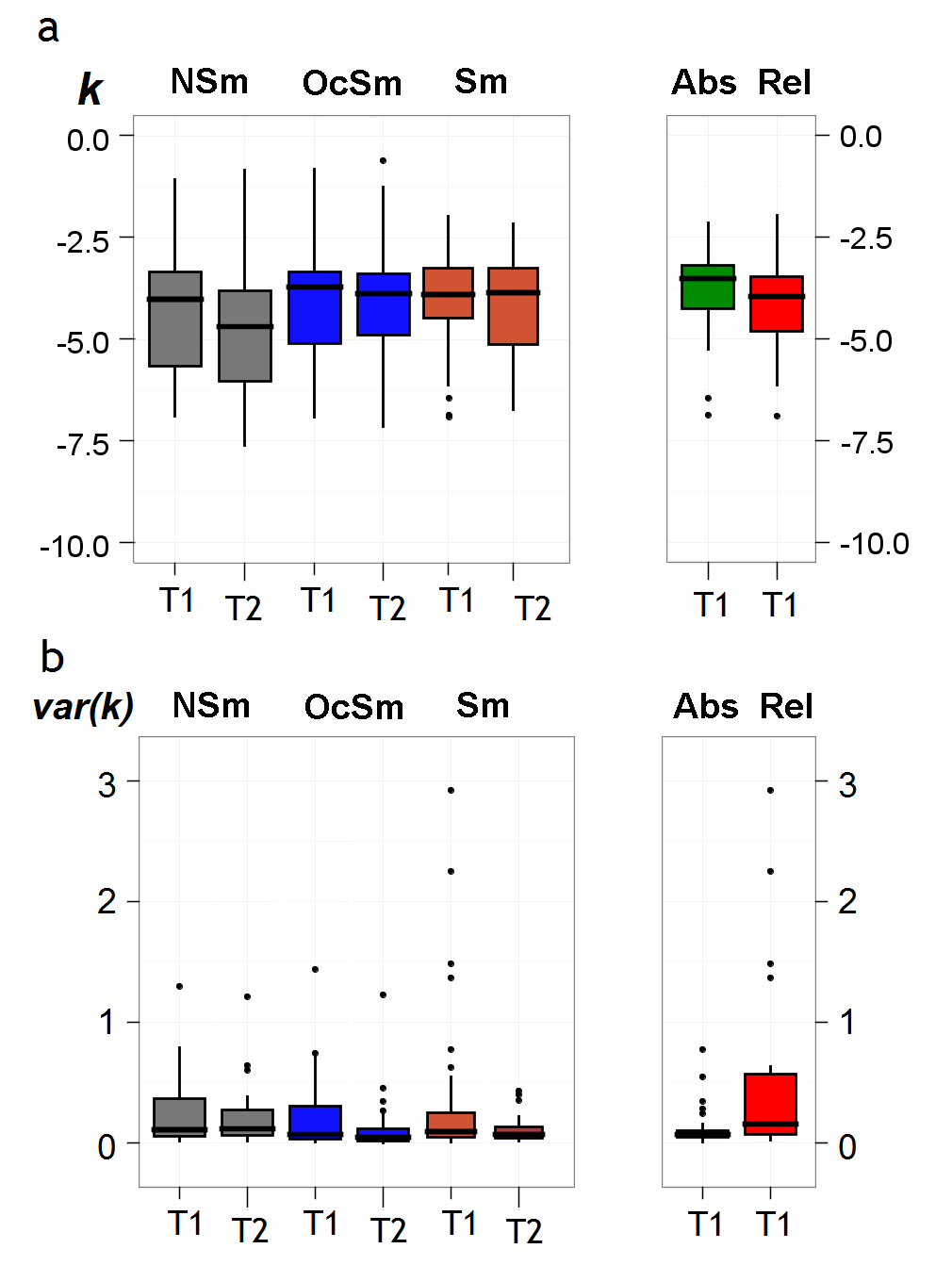


**Fig. S1** Boxplots of *k* and *var(k)* at T1 and T2. (a) No significant group differences between treatment-seeking smokers (brown) and controls (grey: non-smokers, blue: occasional smokers) of delay discounting rate *k* were found. Smoking cessation (mean abstinence of 21 days) did not influence discounting at T2. Yet, one limitation is that smokers who relapsed before their second appointment were not invited and therefore could not be included into T2 group analysis. Baseline discounting rate *k* of smokers relapsing within 30 days (red: relapsers) was not higher compared to those who abstained (green: abstainers). (b) There were no differences in *var(k)* between treatment-seeking smokers (brown) and controls (grey: non-smokers, blue: occasional smokers). However, *var(k)* at T1 was higher in relapsers (red) compared to abstainers (green).
